# Vicarious motor activation during action perception: beyond correlational evidence

**DOI:** 10.3389/fnhum.2013.00185

**Published:** 2013-05-14

**Authors:** Alessio Avenanti, Matteo Candidi, Cosimo Urgesi

**Affiliations:** ^1^Dipartimento di Psicologia, Alma Mater Studiorum, Università di BolognaBologna, Italy; ^2^Centro Studi e Ricerche in Neuroscienze Cognitive, Campus di CesenaCesena, Italy; ^3^Istituto di Ricovero e Cura a Carattere Scientifico Fondazione Santa LuciaRoma, Italy; ^4^Dipartimento di Psicologia, Università “Sapienza” di RomaRome, Italy; ^5^Dipartimento di Scienze Umane, Università di UdineUdine, Italy; ^6^Istituto di Ricovero e Cura a Carattere Scientifico “E. Medea”, Polo Friuli Venezia Giulia, San Vito al TagliamentoPordenone, Italy

**Keywords:** action perception, action simulation, mirror neurons, brain lesion, transcranial magnetic stimulation (TMS)

## Abstract

Neurophysiological and imaging studies have shown that seeing the actions of other individuals brings about the vicarious activation of motor regions involved in performing the same actions. While this suggests a simulative mechanism mediating the perception of others' actions, one cannot use such evidence to make inferences about the functional significance of vicarious activations. Indeed, a central aim in social neuroscience is to comprehend how vicarious activations allow the understanding of other people's behavior, and this requires to use stimulation or lesion methods to establish causal links from brain activity to cognitive functions. In the present work, we review studies investigating the effects of transient manipulations of brain activity or stable lesions in the motor system on individuals' ability to perceive and understand the actions of others. We conclude there is now compelling evidence that neural activity in the motor system is critical for such cognitive ability. More research using causal methods, however, is needed in order to disclose the limits and the conditions under which vicarious activations are required to perceive and understand actions of others as well as their emotions and somatic feelings.

## Vicarious motor activations during action perception

There is now extensive neurophysiological evidence that monkeys—and possibly humans—are equipped with a particular class of neurons active during action execution and action perception (Cattaneo and Rizzolatti, 2009; Mukamel et al., 2010). These so called “mirror neurons” are thought to implement a mechanism matching perceived actions to one's own motor representation of similar actions (di Pellegrino et al., 1992; Gallese et al., 1996; Fogassi et al., 2005). By showing that action perception modulates activity within the motor system, the discovery of mirror neurons has provided direct neurophysiological evidence in favor of the older notion that action perception is inherently linked to action execution (Lotze, 1852; James, 1890; Prinz, 1997). This idea was further supported in humans by functional magnetic resonance imaging (fMRI) (Etzel et al., 2008; Gazzola and Keysers, 2009; Kilner et al., 2009; Oosterhof et al., 2010), electroencephalography (EEG) (Cochin et al., 1999; Lepage and Théoret, 2006; Arnstein et al., 2011), and magnetoencephalography (MEG) (Nishitani and Hari, 2002; Nishitani et al., 2004) evidence that the action observation network (AON), i.e., the neural network activated by seeing others' actions, largely overlaps with the brain network involved in action execution. This has supported the notion that perceiving and understanding others' actions may be based on their vicarious representations within the observer's motor system.

One of the most convincing evidence that action observation vicariously activates motor circuits involved in performing the observed action in humans comes from single-pulse transcranial magnetic stimulation (TMS) studies. This neurophysiological method implies that single magnetic pulses are administered over the participants' primary motor cortex to record motor-evoked potentials from the targeted muscles and assess the excitability of their corticospinal representation under different experimental conditions. Many studies have shown that observing others' actions increases the excitability of the observers' corticospinal motor system (Fadiga et al., 1995; Catmur et al., 2007; Enticott et al., 2010, 2011; Senot et al., 2011). This “motor resonance” appears to be present for both transitive and intransitive movements (Fadiga et al., 2005; Romani et al., 2005; Borgomaneri et al., 2012) and is specific for the muscles involved in the observed action (Urgesi et al., 2006a; Alaerts et al., 2009; Candidi et al., 2010). Moreover, motor resonance is largely automatic and occurs early in time (Lepage et al., 2010; Barchiesi and Cattaneo, 2012). Furthermore, motor resonance is temporally coupled with the observed action when this is dynamically displayed (Gangitano et al., 2004) and seems to encode specific motor features such as the direction (Stefan et al., 2005; Barchiesi and Cattaneo, 2012) and the apparent effort of the action (Alaerts et al., 2010a,b; Tidoni et al., 2013). These findings demonstrate that action observation induces a dynamic simulation of the observed movement in the onlooker's motor system. Studies using cyclic movements (Borroni et al., 2005) or static images of actions (Urgesi et al., 2006b, 2010; Avenanti et al., 2013), however, also indicate that action simulation may be biased toward the future phases of the observed movements, suggesting the motor system is involved in the predictive coding of observed actions as also highlighted by intracortical recordings in monkeys' premotor cortex (Umiltà et al., 2001).

Interestingly, motor resonance appears to be sensitive to higher order aspects of others' actions, such as the goal or the intention of the actor (Cattaneo et al., 2009; Tidoni et al., 2013), suggesting that motor cortex activity is influenced by processing occurring in higher-order regions within the AON. In keeping, there is now direct evidence that resonance in the motor cortex reflects computations carried out in the inferior frontal cortex (IFC, including the ventral premotor cortex and the posterior part of the inferior frontal gyrus) and the inferior parietal lobule (IPL). This is demonstrated by perturb-and-measure studies (Paus, 2005; Avenanti et al., 2007) in which off-line suppression of neural activity in IFC disrupts the motor facilitation induced by action observation (Avenanti et al., 2007, 2013; Enticott et al., 2012) and dual coil studies in which stimulation of IFC and IPL modulates motor cortex reactivity to observed actions (Koch et al., 2010; Catmur et al., 2011).

## Brain stimulation and lesion methods to highlight causal link between AON and action perception

While neurophysiological and brain imaging techniques have been essential in highlighting that action simulation is automatically triggered by action observation, it should be noted that these approaches only provide correlational evidence and cannot establish whether neural activity in motor regions is necessary for action perception. Behavioral studies have shown that action execution affects the perception of others' actions, suggesting a close link between motor and perceptual processing in social interactions (Kilner et al., 2003; Hamilton et al., 2004; Schütz-Bosbach and Prinz, 2007a,b; D'Ausilio et al., 2010; Sacheli et al., 2012, 2013). Motor experts present superior perceptual abilities in the prediction of others' actions (Abernethy et al., 2008; Aglioti et al., 2008) and short-term action execution training improves perception of full (Hecht et al., 2001; Urgesi et al., 2012) and point-light (Casile and Giese, 2006) displays of the same action even if no visual feedback is provided during the execution phase. On the other hand, non-use of specific body parts, following massive deafferentation of lower limbs in spinal cord injury patients, leads to impaired recognition of their movements depicted in static images (Pernigo et al., 2012) and point-light (Arrighi et al., 2011) displays.

While behavioral studies have shown an influence of action execution on action perception these approaches do not tell “where” in the brain these two functions interact. Thus, to test the causal role of specific visuo-motor nodes of the AON in action perception is fundamental to recur to causal methods, i.e., investigating the effect of brain damage or non-invasive brain stimulation of parieto-frontal AON regions on the ability to perceive and recognize others' actions (Avenanti and Urgesi, 2011).

Mounting evidence suggests that IFC and IPL are critical for action perception. In two studies participants were presented with videos of an actor lifting and placing a box that could be of different weights and were asked to estimate the weight of the box (Pobric and Hamilton, 2006) or to recognize whether the actor was trying to provide deceiving information about the weight of the box (Tidoni et al., 2013). It was found that online repetitive TMS over IFC but not over occipital cortex or temporo-parietal junction worsened participants' performance in such tasks that required to monitor spatio-temporal features of seen actions (e.g., arm acceleration). Notably, no change in performance was found in “temporal” control tasks requiring to estimate how long the actor's hand was visible (Pobric and Hamilton, 2006) or in a “spatial” control task requiring to monitor the hand path during lifting and placing (Tidoni et al., 2013). Taken together these studies suggest that IFC is actively involved in processing seen kinematics and in particular in integrating their spatial and temporal features, which may be important to predict others' actions (see also Stadler et al., 2012; Avenanti et al., 2013; Costantini et al., 2013).

The integration of spatio-temporal features is critical for biological-motion perception in order to blend the coherent motion pattern of a series of point-lights into a unitary perception of a moving individual. While voxel-based morphometry (Gilaie-Dotan et al., 2013) and fMRI studies (Saygin et al., 2004) suggest a relation between IFC and biological motion perception, causal methods have recently demonstrated that off-line TMS suppression of IFC activity (van Kemenade et al., 2012) or vascular lesion to IFC (Saygin, 2007) impairs the ability to detect biological motion from point light displays.

Another group of studies has suggested a role of IFC in processing configurational aspects of observed actions (e.g., limb displacement, postures). In such studies participants were presented with static images showing hand grips (Jacquet and Avenanti, 2013), upper or lower limb actions (Urgesi et al., 2007b) or whole body movements (Urgesi et al., 2007a). In all the studies it was found that stimulation of IFC but not of control regions impaired the ability to visually discriminate between pictures depicting two slightly different actions. Notably, brain damage patients with lesions occurring in IFC but not in posterior regions were also impaired in similar tasks (Moro et al., 2008). Interestingly, these impairments in processing configurational aspects of others' actions appear specific for biologically movements because IFC stimulation does not impair visual discrimination of images implying biomechanically impossible body movements (Candidi et al., 2008).

**Figure 1:**
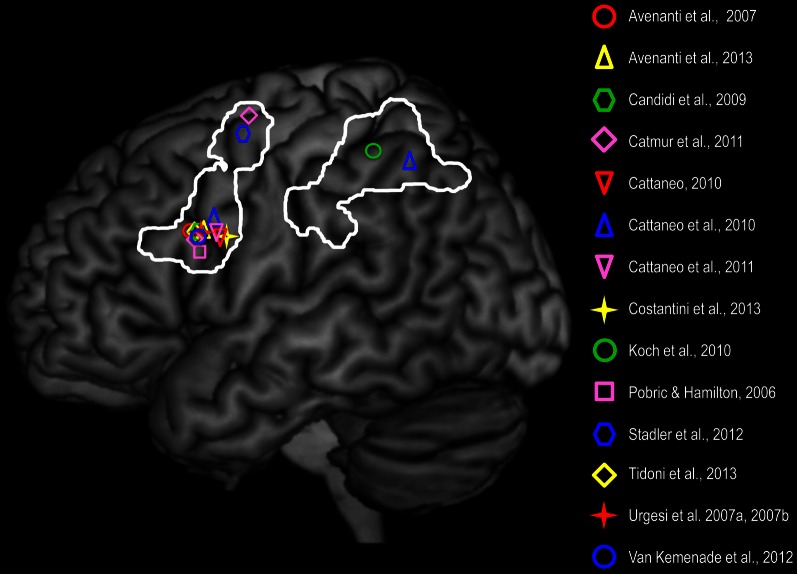
**Frontal and parietal brain sites whose non-invasive stimulation affected: (i) motor resonance, as shown by perturb-and-measure (Avenanti et al., 2007, 2013) and dual coil TMS (Koch et al., 2010; Catmur et al., 2011); (ii) proactive gaze shift during observation of actions toward objects, as shown by virtual lesion (Costantini et al., 2013); and (iii) visual action perception as shown by virtual lesion (Pobric and Hamilton, 2006; Urgesi et al., 2007a,b; Candidi et al., 2008; van Kemenade et al., 2012; Tidoni et al., 2013) and state-dependent TMS (Cattaneo, 2010; Cattaneo et al., 2010, 2011).** In the study of Catmur et al. (2011) IFC and dorsal premotor cortices were stimulated in the right hemisphere but are represented on a left hemisphere. The white lines define frontal (IFC and dorsal premotor) and parietal (IPL and somatosensory) nodes of the AON and are based on a meta-analysis of 139 functional imaging studies investigating action perception (Caspers et al., 2010).

While brain stimulation studies suggest a role of the AON, and of IFC in particular (Figure 1), in processing specific spatio-temporal and configurational features of seen actions, neuropsychological evaluation of brain damage patients shows that lesions in IFC and premotor cortices may lead to more global deficits in action perception and understanding. Lesion in IFC and premotor cortices reduces the ability to: (i) associate pictures of pantomimes (e.g., licking) to the corresponding appropriate object (ice cream) (Saygin et al., 2004); (ii) judge whether a transitive action or an intransitive gesture is correctly performed (Pazzaglia et al., 2008a); (iii) associate the sounds evoking human actions with pictures representing the same actions (Pazzaglia et al., 2008b); (iv) or re-order pictures of human actions compared to physical events (Fazio et al., 2009). On the other hand, lesion of the IPL impairs the recognition of transitive gestures (Buxbaum et al., 2005; Weiss et al., 2008; Kalénine et al., 2010) and of biological motion (Battelli et al., 2003). Moreover, Tranel et al. (2003) showed that patients with lesions in both IFC and IPL were impaired in tasks involving recognition of action from static pictures. Interestingly, there is a specific relation between the motor deficits shown by brain lesion patients and their impairment in action recognition (Eskenazi et al., 2009; Serino et al., 2010). For instance, patients with fronto-parietal lesions who were impaired in performing limb (limb apraxia) or mouth gestures (buccofacial apraxia) were also impaired in the audio-visual matching of hand and mouth actions, respectively (Pazzaglia et al., 2008b). Although the clinical pattern of apraxic patients is complex and cannot be reduced to the dysfunction of the AON visuo-motor nodes, the effector-specific correspondence between their motor and perceptual deficits further hints at the strict link between action execution and perception. In sum, there is now strong evidence that the activation of parieto-frontal nodes of the AON is not merely associated to action observation, but it appears to be critical to perceive and understand the actions of others.

## State-dependent brain stimulation in action perception

One important limitation of causal approaches is that brain damage or non-invasive brain stimulation have remote effects. Although TMS is more focal than other non-invasive brain stimulation methods (i.e., transcranial direct current stimulation), and provides extremely high time-resolution, it modulates activity not only in the neurons under the coil but also in interconnected regions (Ruff et al., 2009; Siebner et al., 2009; Avenanti et al., 2012a,b; Arfeller et al., 2013). Thus, impairment of action perception due to vascular or “virtual,” TMS-induced lesions over specific motor regions may be at least partially due to the disconnection of a larger circuit (i.e., the AON) or the spread of the TMS-induced excitation along its connections (Valero-Cabré et al., 2005, 2007). The simultaneous combination of TMS with functional imaging promises to be of especially great value to tease apart the functional relevance of TMS-induced local and remote neural effects.

Moreover, one should notice that classical virtual lesions approaches do not elucidate how distinct neural populations within the stimulated area interact to give rise to perception and behavior (Silvanto et al., 2008; Avenanti and Urgesi, 2011; Silvanto and Pascual-Leone, 2012). Recently, the TMS-adaptation and TMS-priming paradigms have been developed to tackle such limitation. The paradigms are based on the well-established notion of state-dependency, i.e., that TMS effects depend on the initial state of the stimulated neurons (Lang et al., 2004; Siebner et al., 2004, 2009; Bestmann et al., 2010). In such paradigms the functional state of the neurons is manipulated by means of perceptual (or motor) adaptation or priming. Although the underlying neurophysiological mechanisms are not well understood (Ruzzoli et al., 2011; Schwarzkopf et al., 2011; Perini et al., 2012), the phenomenology of TMS-adaptation and TMS-priming is very robust and consists in a TMS reduction or reverse of the behavioral effects classically induced by perceptual adaptation or priming. These effects unambiguously indicate the presence of neurons encoding for the adapted/primed feature in the stimulated area and their relevance for perceptual processing.

To date, state-dependent TMS has been used to explore perceptual encoding of goal and grip configurations in frontal, parietal, and visual nodes of the AON. For example, in a TMS-priming study of Cattaneo (2010) participants were presented with target pictures showing a hand grasping an object and were asked to judge whether the movement was fast or slow. Observed grasp types varied from precision (index finger and thumb involved only) to whole-hand grasp. Target pictures were preceded by similar prime pictures. Without TMS and with sham stimulation, a clear priming effect was observed as a shortening of reaction times and as a bias toward the priming grasp type in the classification responses. The perceptual advantage of priming was reversed by TMS over IFC, suggesting that distinct populations in such regions are tuned to different observed grasp types and are critical for perception. In a recent TMS-adaptation study, Cattaneo et al. (2010) used perceptual adaptation to goal-directed actions and showed that IFC and IPL contain distinct populations encoding the goal of observed action (i.e., grasping or pulling) independently from the effector (i.e., hand or foot) used to perform such actions. To test whether the same motor neurons involved in performing an action are critical for visual perception of the same action, Cattaneo et al. (2011) used cross-modal motor-to-visual TMS-adaptation. They asked participants to repeatedly perform an action (pushing or pulling) and then to categorize static images showing an actor's hand displacing a ball as pushing or pulling actions. Repeated motor performance induced a visual aftereffect when categorizing action stimuli, with a bias toward pulling after execution of pushing and a bias toward pushing after execution of pulling. Thus, the aftereffect following motor adaptation was a bias toward the action opposite to the one that had been trained, suggesting a motor-to-visual adaptation of the same visuo-motor neurons involved in action execution and observation. Notably, TMS over IFC but not over control regions disrupted such visuo-motor aftereffects. Thus, cross-modal TMS-adaptation provides complimentary evidence to fMRI adaptation studies investigating the attenuation of hemodynamic responses in AON regions after repeated execution and observation of actions. These studies reported action-specific cross-modal adaptation in fronto-parietal AON areas (Chong et al., 2008; Kilner et al., 2009; Lingnau et al., 2009), suggesting the same neural populations are activated in response to specific actions that are either observed or executed. Using the TMS-adaptation paradigm allowed documenting that the same populations of neurons involved in action execution are also critical for action perception.

## Conclusions and future directions

In conclusion, the studies reviewed here provide striking evidence that action perception not only correlates with motor activations in the observer's brain, but also requires these activations for allowing dynamic representations of others' actions. Successful social interactions, however, require motor, sensorial, cognitive, and emotional representations of the behavior of conspecifics. There is now substantial evidence that perceiving the emotions (Carr et al., 2003; Gallese et al., 2004; Dapretto et al., 2006; Bastiaansen et al., 2009) as well as the bodily sensations of others such as touch (Keysers et al., 2004; Blakemore et al., 2005; Bufalari et al., 2007; Ebisch et al., 2008; Schaefer et al., 2009; Gazzola et al., 2012) or pain (Singer et al., 2004, 2006; Avenanti et al., 2005, 2009a; Valeriani et al., 2008; Lamm et al., 2011; Voisin et al., 2011) vicariously activates those brain regions involved in the first hand experience of such emotions and bodily sensations. Although it is held that the mechanism underlying perception of others' sensory or emotional feelings is similar to that underlying action perception (Gallese et al., 2004; Keysers et al., 2010; Gallese and Sinigaglia, 2011), fewer studies have addressed the issue of causality in the former relative to the latter case. However, some of these studies have been important in clarifying that, for example, somatosensory cortices are not only active but are also critical for recognition of others' emotional expressions (Adolphs et al., 2000; Pitcher et al., 2008; Banissy et al., 2010) and others' tactile experiences (Bolognini et al., 2011, 2012, 2013; Rossetti et al., 2012). Further studies, however, are needed to corroborate the causal link between vicarious activations and the understanding of others' sensorial and emotional states.

One critical question for future research concerns the degree to which vicarious activations interact with other mechanism to give rise to perception and understanding of others' actions and feelings. Mirroring and simulating others' actions and feelings may be just one strategy amongst many to gain knowledge of others' mental states. There may be inter-individual differences in the extent to which this strategy is deployed as well as some modulatory effect of social context and previous experience. Vicarious somatomotor activations are often correlated with interindividual differences in personality (Gazzola et al., 2006; Avenanti et al., 2009b; Minio-Paluello et al., 2009; Schaefer et al., 2012) and are influenced by previous experience with the same situation (Calvo-Merino et al., 2006; Cross et al., 2006; Cheng et al., 2007; Fourkas et al., 2008; Abreu et al., 2012; Candidi et al., 2012; Tomeo et al., 2012), and social group belonging (Xu et al., 2009; Avenanti et al., 2010; Hein et al., 2010; Azevedo et al., 2012). They are modulated also by a number of other factors ranging from body ownership (Schütz-Bosbach et al., 2006, 2009) to social tasks and contexts (Kokal et al., 2009; Donne et al., 2011; Sartori et al., 2011). It is thus fundamental to understand the functional significance of such differential activations and causal methods may provide direct information about how and when simulation plays a critical role in our understanding of others' mind.

### Conflict of interest statement

The authors declare that the research was conducted in the absence of any commercial or financial relationships that could be construed as a potential conflict of interest.

## References

[B1] Abernethy B. Zawi K. Jackson R. C. (2008). Expertise and attunement to kinematic constraints. Perception 37, 931–948 18686711 10.1068/p5340

[B2] Abreu A. M. Macaluso E. Azevedo R. T. Cesari P. Urgesi C. Aglioti S. M. (2012). Action anticipation beyond the action observation network: a functional magnetic resonance imaging study in expert basketball players. Eur. J. Neurosci. 35, 1646–1654 10.1111/j.1460-9568.2012.08104.x 22541026

[B3] Adolphs R. Damasio H. Tranel D. Cooper G. Damasio A. R. (2000). A role for somatosensory cortices in the visual recognition of emotions as revealed by three-dimensional lesion mapping. J. Neurosci. 20, 2683–2690 10729349 10.1523/JNEUROSCI.20-07-02683.2000 PMC6772225

[B5] Aglioti S. M. Cesari P. Romani M. Urgesi C. (2008). Action anticipation and motor resonance in elite basketball players. Nat. Neurosci. 11, 1109–1116 19160510 10.1038/nn.2182

[B6] Alaerts K. Heremans E. Swinnen S. P. Wenderoth N. (2009). How are observed actions mapped to the observer's motor system? Influence of posture and perspective. Neuropsychologia 47, 415–422 10.1016/j.neuropsychologia.2008.09.012 18926836

[B7] Alaerts K. Senot P. Swinnen S. P. Craighero L. Wenderoth N. Fadiga L. (2010a). Force requirements of observed object lifting are encoded by the observer's motor system: a TMS study. Eur. J. Neurosci. 31, 1144–1153 10.1111/j.1460-9568.2010.07124.x 20377627

[B8] Alaerts K. Swinnen S. P. Wenderoth N. (2010b). Observing how others lift light or heavy objects: which visual cues mediate the encoding of muscular force in the primary motor cortex? Neuropsychologia 48, 2082–2090 10.1016/j.neuropsychologia.2010.03.029 20381505

[B9] Arfeller C. Schwarzbach J. Ubaldi S. Ferrari P. Barchiesi G. Cattaneo L. (2013). Whole-brain haemodynamic after-effects of 1-Hz magnetic stimulation of the posterior superior temporal cortex during action observation. Brain Topogr. 26, 278–291 10.1007/s10548-012-0239-9 22772359

[B10] Arnstein D. Cui F. Keysers C. Maurits N. M. Gazzola V. (2011). μ-suppression during action observation and execution correlates with BOLD in dorsal premotor, inferior parietal, and SI cortices. J. Neurosci. 31, 14243–14249 10.1523/JNEUROSCI.0963-11.2011 21976509 PMC6623646

[B11] Arrighi R. Cartocci G. Burr D. (2011). Reduced perceptual sensitivity for biological motion in paraplegia patients. Curr. Biol. 21, R910–R911 10.1016/j.cub.2011.09.048 22115454

[B12] Avenanti A. Annella L. Candidi M. Urgesi C. Aglioti S. M. (2013). Compensatory plasticity in the action observation network: virtual lesions of STS enhance anticipatory simulation of seen actions. Cereb. Cortex 23, 570–580 10.1093/cercor/bhs040 22426335

[B13] Avenanti A. Bolognini N. Maravita A. Aglioti S. M. (2007). Somatic and motor components of action simulation. Curr. Biol. 17, 2129–2135 10.1016/j.cub.2007.11.045 18083517

[B14] Avenanti A. Bueti D. Galati G. Aglioti S. M. (2005). Transcranial magnetic stimulation highlights the sensorimotor side of empathy for pain. Nat. Neurosci. 8, 955–960 10.1038/nn1481 15937484

[B15] Avenanti A. Coccia M. Ladavas E. Provinciali L. Ceravolo M. G. (2012a). Low-frequency rTMS promotes use-dependent motor plasticity in chronic stroke: a randomized trial. Neurology 78, 256–264 10.1212/WNL.0b013e3182436558 22238412

[B16] Avenanti A. Annela L. Serino A. (2012b). Suppression of premotor cortex disrupts motor coding of peripersonal space. Neuroimage 63, 281–288 10.1016/j.neuroimage.2012.06.063 22776447

[B17] Avenanti A. Urgesi C. (2011). Understanding ‘what’ others do: mirror mechanisms play a crucial role in action perception. Soc. Cogn. Affect. Neurosci. 6, 257–259 10.1093/scan/nsr004 21653637 PMC3110438

[B18] Avenanti A. Minio-Paluello I. Bufalari I. Aglioti S. M. (2009a). The pain of a model in the personality of an onlooker: influence of state-reactivity and personality traits on embodied empathy for pain. Neuroimage 44, 275–283 10.1016/j.neuroimage.2008.08.001 18761092

[B19] Avenanti A. Minio-Paluello I. Sforza A. Aglioti S. M. (2009b). Freezing or escaping? Opposite modulation of empathic reactivity to the pain of others. Cortex 45, 1072–1076 19100972 10.1016/j.cortex.2008.10.004

[B20] Avenanti A. Sirigu A. Aglioti S. M. (2010). Racial bias reduces empathic sensorimotor resonance with other-race pain. Curr. Biol. 20, 1018–1022 10.1016/j.cub.2010.03.071 20537539

[B21] Azevedo R. T. Macaluso E. Avenanti A. Santangelo V. Cazzato V. Aglioti S. M. (2012). Their pain is not our pain: brain and autonomic correlates of empathic resonance with the pain of same and different race individuals. Hum. Brain Mapp. [Epub ahead of print]. 10.1002/hbm.22133 22807311 PMC6870096

[B22] Banissy M. J. Sauter D. A. Ward J. Warren J. E. Walsh V. Scott S. K. (2010). Suppressing sensorimotor activity modulates the discrimination of auditory emotions but not speaker identity. J. Neurosci. 30, 13552–13557 10.1523/JNEUROSCI.0786-10.2010 20943896 PMC4246058

[B23] Barchiesi G. Cattaneo L. (2012). Early and late motor responses to action observation. Soc. Cogn. Affect. Neurosci. [Epub ahead of print]. 10.1093/scan/nss049 22563004 PMC3739914

[B25] Bastiaansen J. A. Thioux M. Keysers C. (2009). Evidence for mirror systems in emotions. Philos. Trans. R. Soc. Lond. B Biol. Sci. 364, 2391–2404 10.1098/rstb.2009.0058 19620110 PMC2865077

[B26] Battelli L. Cavanagh P. Thornton I. M. (2003). Perception of biological motion in parietal patients. Neuropsychologia 41, 1808–1816 10.1016/S0028-3932(03)00182-9 14527544

[B27] Bestmann S. Swayne O. Blankenburg F. Ruff C. C. Teo J. Weiskopf N. (2010). The role of contralesional dorsal premotor cortex after stroke as studied with concurrent TMS-fMRI. J Neurosci. 30, 11926–11937 10.1523/JNEUROSCI.5642-09.2010 20826657 PMC3044467

[B28] Blakemore S. J. Bristow D. Bird G. Frith C. Ward J. (2005). Somatosensory activations during the observation of touch and a case of vision-touch synaesthesia. Brain 128, 1571–1583 10.1093/brain/awh500 15817510

[B29] Bolognini N. Olgiati E. Xaiz A. Posteraro L. Ferraro F. Maravita A. (2012). Touch to see: neuropsychological evidence of a sensory mirror system for touch. Cereb. Cortex 22, 2055–2064 10.1093/cercor/bhr283 21988827

[B30] Bolognini N. Rossetti A. Convento S. Vallar G. (2013). Understanding others' feelings: the role of the right primary somatosensory cortex in encoding the affective valence of others' touch. J. Neurosci. 33, 4201–4205 10.1523/JNEUROSCI.4498-12.2013 23447627 PMC6619319

[B31] Bolognini N. Rossetti A. Maravita A. Miniussi C. (2011). Seeing touch in the somatosensory cortex: a TMS study of the visual perception of touch. Hum. Brain Mapp. 32, 2104–2114 10.1002/hbm.21172 21305659 PMC6870269

[B32] Borgomaneri S. Gazzola V. Avenanti A. (2012). Motor mapping of implied actions during perception of emotional body language. Brain Stimul. 5, 70–76 10.1016/j.brs.2012.03.011 22503473

[B33] Borroni P. Montagna M. Cerri G. Baldissera F. (2005). Cyclic time course of motor excitability modulation during the observation of a cyclic hand movement. Brain Res. 1065, 115–124 10.1016/j.brainres.2005.10.034 16297887

[B34] Bufalari I. Aprile T. Avenanti A. Di Russo F. Aglioti S. M. (2007). Empathy for pain and touch in the human somatosensory cortex. Cereb. Cortex 17, 2553–2561 10.1093/cercor/bhl161 17205974

[B35] Buxbaum L. J. Kyle K. M. Menon R. (2005). On beyond mirror neurons: internal representations subserving imitation and recognition of skilled object-related actions in humans. Brain Res. Cogn. Brain Res. 25, 226–239 10.1016/j.cogbrainres.2005.05.014 15996857

[B36] Calvo-Merino B. Grèzes J. Glaser D. E. Passingham R. E. Haggard P. (2006). Seeing or doing? Influence of visual and motor familiarity in action observation. Curr. Biol. 16, 1905–1910 10.1016/j.cub.2006.07.065 17027486

[B37] Candidi M. Sacheli L. M. Mega I. Aglioti S. M. (2012). Somatotopic mapping of piano fingering errors in sensorimotor experts: TMS studies in pianists and visually trained musically naïves. Cereb. Cortex. [Epub ahead of print]. 10.1093/cercor/bhs325 23064109

[B38] Candidi M. Urgesi C. Ionta S. Aglioti S. M. (2008). Virtual lesion of ventral premotor cortex impairs visual perception of biomechanically possible but not impossible actions. Soc. Neurosci. 3, 388–400 10.1080/17470910701676269 18979387

[B39] Candidi M. Vicario C. M. Abreu A. N. Aglioti S. M. (2010). Competing mechanisms for mapping action-related categorical knowledge and observed actions. Cereb. Cortex 20, 2832–2841 10.1093/cercor/bhq033 20237242

[B40] Carr L. Iacoboni M. Dubeau M. C. Mazziotta J. C. Lenzi G. L. (2003). Neural mechanisms of empathy in humans: a relay from neural systems for imitation to limbic areas. Proc. Natl. Acad. Sci. U.S.A. 100, 5497–5502 10.1073/pnas.0935845100 12682281 PMC154373

[B41] Casile A. Giese M. A. (2006). Nonvisual motor training influences biological motion perception. Curr. Biol. 16, 69–74 10.1016/j.cub.2005.10.071 16401424

[B42] Caspers S. Zilles K. Laird A. R. Eickhoff S. B. (2010). ALE meta-analysis of action observation and imitation in the human brain. Neuroimage 50, 1148–1167 10.1016/j.neuroimage.2009.12.112 20056149 PMC4981639

[B43] Catmur C. Mars R. B. Rushworth M. F. Heyes C. (2011). Making mirrors: premotor cortex stimulation enhances mirror and counter-mirror motor facilitation. J. Cogn. Neurosci. 23, 2352–2362 10.1162/jocn.2010.21590 20946056

[B44] Catmur C. Walsh V. Heyes C. (2007). Sensorimotor learning configures the human mirror system. Curr. Biol. 17, 1527–1531 10.1016/j.cub.2007.08.006 17716898

[B45] Cattaneo L. (2010). Tuning of ventral premotor cortex neurons to distinct observed grasp types: a TMS-priming study. Exp. Brain Res. 207, 165–172 10.1007/s00221-010-2454-5 20963579

[B46] Cattaneo L. Barchiesi G. Tabarelli D. Arfeller C. Sato M. Glenberg A. M. (2011). One's motor performance predictably modulates the understanding of others' actions through adaptation of premotor visuo-motor neurons. Soc. Cogn. Affect. Neurosci. 6, 301–310 10.1093/scan/nsq099 21186167 PMC3110437

[B47] Cattaneo L. Caruana F. Jezzini A. Rizzolatti G. (2009). Representation of goal and movements without overt motor behavior in the human motor cortex: a transcranial magnetic stimulation study. J. Neurosci. 29, 11134–11138 10.1523/JNEUROSCI.2605-09.2009 19741119 PMC6665924

[B48] Cattaneo L. Rizzolatti G. (2009). The mirror neuron system. Arch. Neurol. 66, 557–560 10.1001/archneurol.2009.41 19433654

[B49] Cattaneo L. Sandrini M. Schwarzbach J. (2010). State-dependent TMS reveals a hierarchical representation of observed acts in the temporal, parietal, and premotor cortices. Cereb. Cortex 20, 2252–2258 10.1093/cercor/bhp291 20051360

[B50] Cheng Y. Lin C. P. Liu H. L. Hsu Y. Y. Lim K. E. Hung D. (2007). Expertise modulates the perception of pain in others. Curr. Biol. 17, 1708–1713 10.1016/j.cub.2007.09.020 17900903

[B51] Chong T. T. Cunnington R. Williams M. A. Kanwisher N. Mattingley J. B. (2008). fMRI adaptation reveals mirror neurons in human inferior parietal cortex. Curr. Biol. 18, 1576–1580 10.1016/j.cub.2008.08.068 18948009 PMC2766090

[B52] Cochin S. Barthelemy C. Roux S. Martineau J. (1999). Observation and execution of movement: similarities demonstrated by quantified electroencephalography. Eur. J. Neurosci. 11, 1839–1842 10.1046/j.1460-9568.1999.00598.x 10215938

[B52a] Costantini M. Ambrosini E. Cardellicchio P. Sinigaglia C. (2013). How your hand drives my eyes. Soc. Cogn. Affect. Neurosci. [Epub ahead of print]. 10.1093/scan/nst037 23559593 PMC4014109

[B53] Cross E. S. Hamilton A. F. Grafton S. T. (2006). Building a motor simulation *de novo*: observation of dance by dancers. Neuroimage 31, 1257–1267 10.1016/j.neuroimage.2006.01.033 16530429 PMC1821082

[B54] Dapretto M. Davies M. S. Pfeifer J. H. Scott A. A. Sigman M. Bookheimer S. Y. (2006). Understanding emotions in others: mirror neuron dysfunction in children with autism spectrum disorders. Nat. Neurosci. 9, 28–30 10.1038/nn1611 16327784 PMC3713227

[B55] D'Ausilio A. Brunetti R. Delogu F. Santonico C. Belardinelli M. O. (2010). How and when auditory action effects impair motor performance. Exp. Brain Res. 201, 323–330 10.1007/s00221-009-2044-6 19847407

[B56] di Pellegrino G. Fadiga L. Fogassi L. Gallese V. Rizzolatti G. (1992). Understanding motor events: a neurophysiological study. Exp. Brain Res. 91, 176–180 1301372 10.1007/BF00230027

[B58] Donne C. M. Enticott P. G. Rinehart N. J. Fitzgerald P. B. (2011). A transcranial magnetic stimulation study of corticospinal excitability during the observation of meaningless, goal-directed, and social behaviour. Neurosci. Lett. 489, 57–61 10.1016/j.neulet.2010.11.067 21134417

[B59] Ebisch S. J. Perrucci M. G. Ferretti A. Del Gratta C. Romani G. L. Gallese V. (2008). The sense of touch: embodied simulation in a visuotactile mirroring mechanism for observed animate or inanimate touch. J. Cogn. Neurosci. 20, 1611–1623 10.1162/jocn.2008.20111 18345991

[B60] Enticott P. G. Arnold S. L. Fitzgibbon B. M. Hoy K. E. Susilo D. A. Fitzgerald P. B. (2012). Transcranial direct current stimulation (tDCS) of the inferior frontal gyrus disrupts interpersonal motor resonance. Neuropsychologia 50, 1628–1631 10.1016/j.neuropsychologia.2012.03.016 22465862

[B61] Enticott P. G. Kennedy H. A. Bradshaw J. L. Rinehart N. J. Fitzgerald P. B. (2010). Understanding mirror neurons: evidence for enhanced corticospinal excitability during the observation of transitive but not intransitive hand gestures. Neuropsychologia 48, 2675–2680 10.1016/j.neuropsychologia.2010.05.014 20470809

[B62] Enticott P. G. Kennedy H. A. Bradshaw J. L. Rinehart N. J. Fitzgerald P. B. (2011). Motor corticospinal excitability during the observation of interactive hand gestures. Brain Res. Bull. 85, 89–95 10.1016/j.brainresbull.2011.03.018 21457764

[B63] Eskenazi T. Grosjean M. Humphreys G. W. Knoblich G. (2009). The role of motor simulation in action perception: a neuropsychological case study. Psychol. Res. 73, 477–485 10.1007/s00426-009-0231-5 19350271 PMC2694935

[B64] Etzel J. A. Gazzola V. Keysers C. (2008). Testing simulation theory with cross-modal multivariate classification of fMRI data. PLoS ONE 3:e3690 10.1371/journal.pone.0003690 18997869 PMC2577733

[B65] Fadiga L. Craighero L. Olivier E. (2005). Human motor cortex excitability during the perception of others' action. Curr. Opin. Neurobiol. 15, 213–218 10.1016/j.conb.2005.03.013 15831405

[B66] Fadiga L. Fogassi L. Pavesi G. Rizzolatti G. (1995). Motor facilitation during action observation: a magnetic stimulation study. J. Neurophysiol. 73, 2608–2611 7666169 10.1152/jn.1995.73.6.2608

[B67] Fazio P. Cantagallo A. Craighero L. D'Ausilio A. Roy A. C. Pozzo T. (2009). Encoding of human action in Broca's area. Brain 132, 1980–1988 10.1093/brain/awp118 19443630

[B68] Fogassi L. Ferrari P. F. Gesierich B. Rozzi S. Chersi F. Rizzolatti G. (2005). Parietal lobe: from action organization to intention understanding. Science 308, 662–667 10.1126/science.1106138 15860620

[B69] Fourkas A. D. Bonavolontà V. Avenanti A. Aglioti S. M. (2008). Kinesthetic imagery and tool-specific modulation of corticospinal representations in expert tennis players. Cereb. Cortex 18, 2382–2390 10.1093/cercor/bhn005 18296436

[B70] Gallese V. Sinigaglia C. (2011). What is so special about embodied simulation? Trends Cogn. Sci. 15, 512–519 10.1016/j.tics.2011.09.003 21983148

[B71] Gallese V. Fadiga L. Fogassi L. Rizzolatti G. (1996). Action recognition in the premotor cortex. Brain 119, 593–609 10.1093/brain/119.2.593 8800951

[B72] Gallese V. Keysers C. Rizzolatti G. (2004). A unifying view of the basis of social cognition. Trends Cogn. Sci. 8, 396–403 10.1016/j.tics.2004.07.002 15350240

[B73] Gangitano M. Mottaghy F. M. Pascual-Leone A. (2004). Modulation of premotor mirror neuron activity during observation of unpredictable grasping movements. Eur. J. Neurosci. 20, 2193–2202 10.1111/j.1460-9568.2004.03655.x 15450099

[B74] Gazzola V. Keysers C. (2009). The observation and execution of actions share motor and somatosensory voxels in all tested subjects: single-subject analyses of unsmoothed fMRI data. Cereb. Cortex 19, 1239–1255 10.1093/cercor/bhn181 19020203 PMC2677653

[B75] Gazzola V. Aziz-Zadeh L. Keysers C. (2006). Empathy and the somatotopic auditory mirror system in humans. Curr. Biol. 16, 1824–1829 10.1016/j.cub.2006.07.072 16979560

[B76] Gazzola V. Spezio M. L. Etzel J. A. Castelli F. Adolphs R. Keysers C. (2012). Primary somatosensory cortex discriminates affective significance in social touch. Proc. Natl. Acad. Sci. U.S.A. 109, E1657–E1666 10.1073/pnas.1113211109 22665808 PMC3382530

[B76a] Gilaie-Dotan S. Kanai R. Bahrami B. Rees G. Saygin A. P. (2013). Neuroanatomical correlates of biological motion detection. Neuropsychologia 51, 457–463 10.1016/j.neuropsychologia.2012.11.027 23211992 PMC3611598

[B77] Hamilton A. Wolpert D. Frith U. (2004). Your own action influences how you perceive another person's action. Curr. Biol. 14, 493–498 10.1016/j.cub.2004.03.007 15043814

[B78] Hecht H. Vogt S. Prinz W. (2001). Motor learning enhances perceptual judgment: a case for action-perception transfer. Psychol. Res. 65, 3–14 11505611 10.1007/s004260000043

[B79] Hein G. Silani G. Preuschoff K. Batson C. D. Singer T. (2010). Neural responses to ingroup and outgroup members' suffering predict individual differences in costly helping. Neuron 68, 149–160 10.1016/j.neuron.2010.09.003 20920798

[B80] Jacquet P. Avenanti A. (2013). Perturbing the action observation network during perception and categorization of actions' goals and grips: state-dependency and virtual lesion TMS effects. Cereb. Cortex (in press). 10.1093/cercor/bht242 24084126

[B81] James W. (1890). The Principles of Psychology. 2 vols. New York, NY: Henry Holt & Co.

[B82] Kalénine S. Buxbaum L. J. Coslett H. B. (2010). Critical brain regions for action recognition: lesion symptom mapping in left hemisphere stroke. Brain 133, 3269–3280 10.1093/brain/awq210 20805101 PMC2965423

[B83] Keysers C. Kaas J. H. Gazzola V. (2010). Somatosensation in social perception. Nat. Rev. Neurosci. 11, 417–428 10.1038/nrn2833 20445542

[B84] Keysers C. Wicker B. Gazzola V. Anton J. L. Fogassi L. Gallese V. (2004). A touching sight: SII/PV activation during the observation and experience of touch. Neuron 42, 335–346 10.1016/S0896-6273(04)00156-4 15091347

[B85] Kilner J. M. Neal A. Weiskopf N. Friston K. J. Frith C. D. (2009). Evidence of mirror neurons in human inferior frontal gyrus. J. Neurosci. 29, 10153–10159 10.1523/JNEUROSCI.2668-09.2009 19675249 PMC2788150

[B86] Kilner J. M. Paulignan Y. Blakemore S. J. (2003). An interference effect of observed biological movement on action. Curr. Biol. 13, 522–525 10.1016/S0960-9822(03)00165-9 12646137

[B87] Koch G. Versace V. Bonnì S. Lupo F. Lo Gerfo E. Oliveri M. (2010). Resonance of cortico-cortical connections of the motor system with the observation of goal directed grasping movements. Neuropsychologia 48, 3513–3520 10.1016/j.neuropsychologia.2010.07.037 20691198

[B88] Kokal I. Gazzola V. Keysers C. (2009). Acting together in and beyond the mirror neuron system. Neuroimage 47, 2046–2056 10.1016/j.neuroimage.2009.06.010 19524043

[B89] Lamm C. Decety J. Singer T. (2011). Meta-analytic evidence for common and distinct neural networks associated with directly experienced pain and empathy for pain. Neuroimage 54, 2492–2502 10.1016/j.neuroimage.2010.10.014 20946964

[B89a] Lang N. Siebner H. R. Ernst D. Nitsche M. A. Paulus W. Lemon R. N. (2004). Preconditioning with transcranial direct current stimulation sensitizes the motor cortex to rapid-rate transcranial magnetic stimulation and controls the direction of after-effects. Biol. Psychiatry 56, 634–639 10.1016/j.biopsych.2004.07.017 15522246

[B90] Lepage J. F. Théoret H. (2006). EEG evidence for the presence of an action observation-execution matching system in children. Eur. J. Neurosci. 23, 2505–2510 10.1111/j.1460-9568.2006.04769.x 16706857

[B91] Lepage J. F. Tremblay S. Théoret H. (2010). Early non specific modulation of corticospinal excitability during action observation. Eur. J. Neurosci. 31, 931–937 10.1111/j.1460-9568.2010.07121.x 20374291

[B92] Lingnau A. Gesierich B. Caramazza A. (2009). Asymmetric fMRI adaptation reveals no evidence for mirror neurons in humans. Proc. Natl. Acad. Sci. U.S.A. 106, 9925–9930 10.1073/pnas.0902262106 19497880 PMC2701024

[B93] Lotze R. H. (1852). Medicinische Psychologie, order, Physiologie der Seele. Leipzig: Weidmann'sche Buchhandlung

[B94] Minio-Paluello I. Baron-Cohen S. Avenanti A. Walsh V. Aglioti S. M. (2009). Absence of embodied empathy during pain observation in Asperger syndrome. Biol. Psychiatry 65, 55–62 10.1016/j.biopsych.2008.08.006 18814863

[B95] Moro V. Urgesi C. Pernigo S. Lanteri P. Pazzaglia M. Aglioti S. M. (2008). The neural basis of body form and body action agnosia. Neuron 60, 235–246 10.1016/j.neuron.2008.09.022 18957216

[B96] Mukamel R. Ekstrom A. Kaplan J. Iacoboni M. Fried I. (2010). Single-neuron responses in humans during execution and observation of actions. Curr. Biol. 20, 750–756 10.1016/j.cub.2010.02.045 20381353 PMC2904852

[B97] Nishitani N. Hari R. (2002). Viewing lip forms: cortical dynamics. Neuron 36, 1211–1220 10.1016/S0896-6273(02)01089-9 12495633

[B98] Nishitani N. Avikainen S. Hari R. (2004). Abnormal imitation-related cortical activation sequences in Asperger's syndrome. Ann. Neurol. 55, 558–562 10.1002/ana.20031 15048895

[B99] Oosterhof N. N. Wiggett A. J. Diedrichsen J. Tipper S. P. Downing P. E. (2010). Surface-based information mapping reveals crossmodal vision-action representations in human parietal and occipitotemporal cortex. J. Neurophysiol. 104, 1077–1089 10.1152/jn.00326.2010 20538772 PMC2934920

[B100] Paus T. (2005). Inferring causality in brain images: a perturbation approach. Philos. Trans. R. Soc. Lond. B Biol. Sci. 360, 1109–1114 10.1098/rstb.2005.1652 16087451 PMC1854935

[B101] Pazzaglia M. Pizzamiglio L. Pes E. Aglioti S. M. (2008a). The sound of actions in apraxia. Curr. Biol. 18, 1766–1772 10.1016/j.cub.2008.09.061 19013068

[B102] Pazzaglia M. Smania N. Corato E. Aglioti S. M. (2008b). Neural underpinnings of gesture discrimination in patients with limb apraxia. J. Neurosci. 28, 3030–3041 10.1523/JNEUROSCI.5748-07.2008 18354006 PMC6670701

[B103] Perini F. Cattaneo L. Carrasco M. Schwarzbach J. V. (2012). Occipital transcranial magnetic stimulation has an activity-dependent suppressive effect. J. Neurosci. 32, 12361–12365 10.1523/JNEUROSCI.5864-11.2012 22956826 PMC3472806

[B104] Pernigo S. Moro V. Avesani R. Miatello C. Urgesi C. Aglioti S. M. (2012). Massive somatic deafferentation and motor deefferentation of the lower part of the body impair its visual recognition: a psychophysical study of patients with spinal cord injury. Eur. J. Neurosci. 36, 3509–3518 10.1111/j.1460-9568.2012.08266.x 22928907

[B105] Pitcher D. Garrido L. Walsh V. Duchaine B. C. (2008). Transcranial magnetic stimulation disrupts the perception and embodiment of facial expressions. J. Neurosci. 28, 8929–8933 10.1523/JNEUROSCI.1450-08.2008 18768686 PMC6670866

[B106] Pobric G. Hamilton A. F. (2006). Action understanding requires the left inferior frontal cortex. Curr. Biol. 16, 524–529 10.1016/j.cub.2006.01.033 16527749

[B107] Prinz W. (1997). Perception and action planning. Eur. J. Cogn. Psychol. 9, 129–154

[B108] Romani M. Cesari P. Urgesi C. Facchini S. Aglioti S. M. (2005). Motor facilitation of the human cortico-spinal system during observation of bio-mechanically impossible movements. Neuroimage 26, 755–763 10.1016/j.neuroimage.2005.02.027 15955484

[B109] Rossetti A. Miniussi C. Maravita A. Bolognini N. (2012). Visual perception of bodily interactions in the primary somatosensory cortex. Eur. J. Neurosci. 36, 2317–2323 10.1111/j.1460-9568.2012.08137.x 22626449

[B110] Ruff C. C. Driver J. Bestmann S. (2009). Combining TMS and fMRI: from ‘virtual lesions’ to functional-network accounts of cognition. Cortex 45, 1043–1049 10.1016/j.cortex.2008.10.012 19166996 PMC2726131

[B111] Ruzzoli M. Abrahamyan A. Clifford C. W. Marzi C. A. Miniussi C. Harris J. A. (2011). The effect of TMS on visual motion sensitivity: an increase in neural noise or a decrease in signal strength? J. Neurophysiol. 106, 138–143 10.1152/jn.00746.2010 21543749

[B112] Sacheli L. M. Candidi M. Pavone E. F. Tidoni E. Aglioti S. M. (2012). And yet they act together: interpersonal perception modulates visuo-motor interference and mutual adjustments during a joint-grasping task. PLoS ONE 7:e50223 10.1371/journal.pone.0050223 23209680 PMC3509140

[B113] Sacheli L. M. Tidoni E. Pavone E. F. Aglioti S. M. Candidi M. (2013). Kinematics fingerprints of Leader and Follower role-taking during cooperative joint actions. Exp. Brain Res. 226, 473–486 10.1007/s00221-013-3459-7 23503771

[B114] Sartori L. Cavallo A. Bucchioni G. Castiello U. (2011). Corticospinal excitability is specifically modulated by the social dimension of observed actions. Exp. Brain Res. 211, 557–568 10.1007/s00221-011-2650-y 21472443

[B115] Saygin A. P. (2007). Superior temporal and premotor brain areas necessary for biological motion perception. Brain 130, 2452–2461 10.1093/brain/awm162 17660183

[B116] Saygin A. P. Wilson S. M. Dronkers N. F. Bates E. (2004). Action comprehension in aphasia: linguistic and non-linguistic deficits and their lesion correlates. Neuropsychologia 42, 1788–1804 10.1016/j.neuropsychologia.2004.04.016 15351628

[B117] Schaefer M. Heinze H. J. Rotte M. (2012). Embodied empathy for tactile events: Interindividual differences and vicarious somatosensory responses during touch observation. Neuroimage 60, 952–957 10.1016/j.neuroimage.2012.01.112 22306799

[B118] Schaefer M. Xu B. Flor H. Cohen L. G. (2009). Effects of different viewing perspectives on somatosensory activations during observation of touch. Hum. Brain Mapp. 30, 2722–2730 10.1002/hbm.20701 19172650 PMC6870795

[B119] Schütz-Bosbach S. Avenanti A. Aglioti S. M. Haggard P. (2009). Don't do it! Cortical inhibition and self-attribution during action observation. J. Cogn. Neurosci. 21, 1215–1227 10.1162/jocn.2009.21068 18702585

[B119a] Schütz-Bosbach S. Mancini B. Aglioti S. M. Haggard P. (2006). Self and other in the human motor system. Curr. Biol. 16, 1830–1834 10.1016/j.cub.2006.07.048 16979561

[B120] Schütz-Bosbach S. Prinz W. (2007a). Prospective coding in event representation. Cogn. Process. 8, 93–102 10.1007/s10339-007-0167-x 17406917

[B121] Schütz-Bosbach S. Prinz W. (2007b). Perceptual resonance: action-induced modulation of perception. Trends Cogn. Sci. 11, 349–355 10.1016/j.tics.2007.06.005 17629544

[B122] Schwarzkopf D. S. Silvanto J. Rees G. (2011). Stochastic resonance effects reveal the neural mechanisms of transcranial magnetic stimulation. J. Neurosci. 31, 3143–3147 10.1523/JNEUROSCI.4863-10.2011 21368025 PMC3059801

[B123] Senot P. D'Ausilio A. Franca M. Caselli L. Craighero L. Fadiga L. (2011). Effect of weight-related labels on corticospinal excitability during observation of grasping: a TMS study. Exp. Brain Res. 211, 161–167 10.1007/s00221-011-2635-x 21533701

[B124] Serino A. De Filippo L. Casavecchia C. Coccia M. Shiffrar M. Làdavas E. (2010). Lesions to the motor system affect action perception. J. Cogn. Neurosci. 22, 413–426 10.1162/jocn.2009.21206 19302003

[B125] Siebner H. R. Hartwigsen G. Kassuba T. Rothwell J. C. (2009). How does transcranial magnetic stimulation modify neuronal activity in the brain? Implications for studies of cognition. Cortex 45, 1035–1042 10.1016/j.cortex.2009.02.007 19371866 PMC2997692

[B125a] Siebner H. R. Lang N. Rizzo V. Nitsche M. A. Paulus W. Lemon R. N. (2004). Preconditioning of low-frequency repetitive transcranial magnetic stimulation with transcranial direct current stimulation: evidence for homeostatic plasticity in the human motor cortex. J. Neurosci. 24, 3379–3385 10.1523/JNEUROSCI.5316-03.2004 15056717 PMC6730024

[B126] Silvanto J. Muggleton N. Walsh V. (2008). State-dependencyinbrainstimulation studies of perception and cognition. Trends Cogn. Sci. 12, 447–454 10.1016/j.tics.2008.09.004 18951833

[B127] Silvanto J. Pascual-Leone A. (2012). Why the assessment of causality in brain-behavior relations requires brain stimulation. J. Cogn. Neurosci. 24, 775–777 10.1162/jocn_a_00193 22264196

[B128] Singer T. Seymour B. O'Doherty J. Kaube H. Dolan R. J. Frith C. D. (2004). Empathy for pain involves the affective but not sensory components of pain. Science 303, 1157–1162 10.1126/science.1093535 14976305

[B129] Singer T. Seymour B. O'Doherty J. P. Stephan K. E. Dolan R. J. Frith C. D. (2006). Empathic neural responses are modulated by the perceived fairness. Nature 439, 466–469 10.1038/nature04271 16421576 PMC2636868

[B129a] Stadler W. Ott D. V. Springer A. Schubotz R. I. Schütz-Bosbach S. Prinz W. (2012). Repetitive TMS suggests a role of the human dorsal premotor cortex in action prediction. Front. Hum. Neurosci. 6:20 10.3389/fnhum.2012.00020 22363279 PMC3282473

[B130] Stefan K. Cohen L. G. Duque J. Mazzocchio R. Celnik P. Sawaki L. (2005). Formation of a motor memory by action observation. J. Neurosci. 25, 9339–9346 10.1523/JNEUROSCI.2282-05.2005 16221842 PMC6725701

[B131] Tidoni E. Borgomaneri S. di Pellegrino G. Avenanti A. (2013). Action simulation plays a critical role in deceptive action recognition. J. Neurosci. 33, 611–623 10.1523/JNEUROSCI.2228-11.2013 23303940 PMC6704902

[B132] Tomeo E. Cesari P. Aglioti S. M. Urgesi C. (2012). Fooling the kickers but not the goalkeepers: behavioral and neurophysiological correlates of fake action detection in soccer. Cereb. Cortex. [Epub ahead of print]. 10.1093/cercor/bhs279 22941722

[B133] Tranel D. Kemmerer D. Adolphs R. Damasio H. Damasio A. R. (2003). Neural correlates of conceptual knowledge for actions. Cogn. Neuropsychol. 20, 409–432 10.1080/02643290244000248 20957578

[B134] Umiltà M. A. Kohler E. Gallese V. Fogassi L. Fadiga L. Keysers C. (2001). I know what you are doing. A neurophysiological study. Neuron 31, 155–165 10.1016/S0896-6273(01)00337-3 11498058

[B135] Urgesi C. Calvo-Merino B. Haggard P. Aglioti S. M. (2007a). Transcranial magnetic stimulation reveals two cortical pathways for visual body processing. J. Neurosci. 27, 8023–8030 10.1523/JNEUROSCI.0789-07.2007 17652592 PMC6672742

[B137] Urgesi C. Candidi M. Ionta S. Aglioti S. M. (2007b). Representation of body identity and body actions in extrastriate body area and ventral premotor cortex. Nat. Neurosci. 10, 30–31 10.1038/nn1815 17159990

[B136] Urgesi C. Candidi M. Fabbro F. Romani M. Aglioti S. M. (2006a). Motor facilitation during action observation: topographic mapping of the target muscle and influence of the onlooker's posture. Eur. J. Neurosci. 23, 2522–2530 10.1111/j.1460-9568.2006.04772.x 16706859

[B139] Urgesi C. Moro V. Candidi M. Aglioti S. M. (2006b). Mapping implied body actions in the human motor system. J. Neurosci. 26, 7942–7949 10.1523/JNEUROSCI.1289-06.2006 16870739 PMC6674209

[B138] Urgesi C. Maieron M. Avenanti A. Tidoni E. Fabbro F. Aglioti S. M. (2010). Simulating the future of actions in the human corticospinal system. Cereb. Cortex 20, 2511–2521 10.1093/cercor/bhp292 20051359

[B140] Urgesi C. Savonitto M. M. Fabbro F. Aglioti S. M. (2012). Long- and short-term plastic modeling of action prediction abilities in volleyball. Psychol. Res. 76, 542–560 10.1007/s00426-011-0383-y 22045443

[B141] Valeriani M. Betti V. Le Pera D. De Armas L. Miliucci R. Restuccia D. (2008). Seeing the pain of others while being in pain: a laser evoked potentials study. Neuroimage 40, 1419–1428 10.1016/j.neuroimage.2007.12.056 18291679

[B142] Valero-Cabré A. Payne B. R. Pascual-Leone A. (2007). Opposite impact on 14C-2-deoxyglucose brain metabolism following patterns of high and low frequency repetitive transcranial magnetic stimulation in the posterior parietal cortex. Exp. Brain Res. 176, 603–615 10.1007/s00221-006-0639-8 16972076

[B143] Valero-Cabré A. Payne B. R. Rushmore J. Lomber S. G. Pascual-Leone A. (2005). Impact of repetitive transcranial magnetic stimulation of the parietal cortex on metabolic brain activity: a 14C-2DG tracing study in the cat. Exp. Brain Res. 163, 1–12 10.1007/s00221-004-2140-6 15688174

[B144] van Kemenade B. M. Muggleton N. Walsh V. Saygin A. P. (2012). Effects of TMS over premotor and superior temporal cortices on biological motion perception. J. Cogn. Neurosci. 24, 896–904 10.1162/jocn_a_00194 22264195

[B145] Voisin J. I. Marcoux L. A. Canizales D. L. Mercier C. Jackson P. L. (2011). I am touched by your pain: limb-specific modulation of the cortical response to a tactile stimulation during pain observation. J. Pain 12, 1182–1189 10.1016/j.jpain.2011.06.005 21911315

[B146] Weiss P. H. Rahbari N. N. Hesse M. D. Fink G. R. (2008). Deficient sequencing of pantomimes in apraxia. Neurology 70, 834–840 10.1212/01.wnl.0000297513.78593.dc 18332341

[B147] Xu X. Zuo X. Wang X. Han S. (2009). Do you feel my pain? Racial group membership modulates empathic neural responses. J. Neurosci. 29, 8525–8529 10.1523/JNEUROSCI.2418-09.2009 19571143 PMC6665679

